# Activation of NK Cells by an Endocytosed Receptor for Soluble HLA-G

**DOI:** 10.1371/journal.pbio.0040009

**Published:** 2005-12-27

**Authors:** Sumati Rajagopalan, Yenan T Bryceson, Shanmuga P Kuppusamy, Daniel E Geraghty, Arnold van der Meer, Irma Joosten, Eric O Long

**Affiliations:** 1Laboratory of Immunogenetics, National Institute of Allergy and Infectious Diseases, National Institutes of Health, Rockville, Maryland, United States of America; **2**Fred Hutchinson Cancer Research Center, Seattle, Washington, United States of America; **3**Department of Blood Transfusion and Transplantation Immunology, Radboud University Nijmegen Medical Centre, Nijmegen, the Netherlands; Harvard Medical SchoolUnited States of America

## Abstract

Signaling from endosomes is emerging as a mechanism by which selected receptors provide sustained signals distinct from those generated at the plasma membrane. The activity of natural killer (NK) cells, which are important effectors of innate immunity and regulators of adaptive immunity, is controlled primarily by receptors that are at the cell surface. Here we show that cytokine secretion by resting human NK cells is induced by soluble, but not solid-phase, antibodies to the killer cell immunoglobulin-like receptor (KIR) 2DL4, a receptor for human leukocyte antigen (HLA)-G. KIR2DL4 was constitutively internalized into Rab5-positive compartments via a dynamin-dependent process. Soluble HLA-G was endocytosed into KIR2DL4–containing compartments in NK cells and in 293T cells transfected with KIR2DL4. Chemokine secretion induced by KIR2DL4 transfection into 293T cells occurred only with recombinant forms of KIR2DL4 that trafficked to endosomes. The profile of genes up-regulated by KIR2DL4 engagement on resting NK cells revealed a proinflammatory/proangiogenic response. Soluble HLA-G induced secretion of a similar set of cytokines and chemokines. This unique stimulation of resting NK cells by soluble HLA-G, which is endocytosed by KIR2DL4, implies that NK cells may provide useful functions at sites of HLA-G expression, such as promotion of vascularization in maternal decidua during early pregnancy.

## Abstract 2

KIR2DL4, a human killer cell immunoglobulin receptor expressed on natural killer cells, can be stimulated by soluble antibody or the soluble version of its natural ligand (HLA-G), and may signal from within endosomes.

## Introduction

Natural killer (NK) cells are a subset of lymphocytes that mediate innate immunity and regulate adaptive immunity via cytokine secretion and cytotoxic activity. The activation of NK cell function is a result of the integration of activating and inhibitory signals delivered by NK cell receptors [1,2]. NK cell receptors recognize ligands that are either up-regulated or expressed constitutively on target cells. Cells can become sensitive to NK cell–mediated cytotoxicity for various reasons, such as loss of major histocompatibility complex (MHC) class I expression or up-regulation of surface molecules in response to DNA damage or stress, which can occur as a result of infection or transformation [3,4]. Normal cells are resistant to NK cell-mediated cytotoxicity due to the presence of inhibitory receptors on the surface of NK cells that recognize major histocompatibility complex (MHC) class I molecules. The interaction between inhibitory receptors and their MHC ligands on target cells transduces a negative signal that blocks the lytic activity of NK cells. NK cells in peripheral blood represent 5%–10% of circulating lymphocytes. In contrast, NK cells are the predominant lymphocyte subset in uterus. In early pregnancy, uterine NK cells proliferate and remain in the decidua basalis, which consists of uterine tissue at the maternal–fetal interface [5]. The precise function of uterine NK cells is still unknown.

Both peripheral blood and uterine human NK cells express killer cell immunoglobulin-like receptors (KIR), a family of NK cell receptors that recognize MHC class I molecules. KIR are type I transmembrane glycoproteins with two or three Ig-like domains and cytoplasmic tails of varying lengths [6]. KIRs with long cytoplasmic domains (KIR2DL and KIR3DL) are inhibitory receptors that contain cytoplasmic immunoreceptor tyrosine-based inhibition motifs (ITIM). Those KIRs with short cytoplasmic domains (KIR2DS and KIR3DS) are activating receptors that associate with the adapter DAP12 via a lysine residue in their transmembrane region. With one exception, KIR genes are not expressed in all NK cells. Rather, each NK cell expresses its own repertoire of KIR genes.

*KIR2DL4* is an evolutionarily conserved, framework member of the *KIR* gene family that is expressed by all KIR haplotypes and in all NK cells. In contrast to other activating or inhibitory KIR family members, which regulate NK cell cytotoxicity and cytokine production, KIR2DL4 activates cytokine production, but not cytotoxicity, in resting NK cells from peripheral blood [7]. It is unique in its genomic organization and regulation and in its protein structure and function [7–12]. *KIR2DL4* is polymorphic with two reported genetic variants, designated *10A* and *9A* [13]. While the product of the *10A* allele is detectable on the cell surface, the *9A* allele, encoding a protein with a truncated cytoplasmic tail, is not stable at the cell surface [14]. KIR2DL4 has a charged arginine residue in its transmembrane region; however, unlike other activating KIR2DS that pair with the adapter DAP-12, KIR2DL4 can associate with the FcRI γ chain [15]. Engagement of KIR2DL4 results in activation despite the inhibitory potential conferred by the presence of an ITIM in its cytoplasmic tail [7,16,17]. In resting, peripheral blood NK cells, ligation of KIR2DL4 with monoclonal antibody (mAb) results in interferon (IFN)-γ production but not cytotoxicity [7]. The very low cell surface expression of KIR2DL4 [14,17] has been difficult to reconcile with the functional outcome associated with this receptor.

Data suggest that KIR2DL4 binds the nonclassical class I molecule HLA-G [8,9]. HLA-G, a nonclassical class I molecule of limited polymorphism, has a unique expression pattern restricted mainly to trophoblast cells that invade the maternal decidua during early pregnancy [18]. HLA-G expression may be inducible in other cell types, in response to inflammation, infection, and transformation [19]. Several isoforms of HLA-G are expressed in the placenta, including membrane-bound forms (HLA-G1, -G2, -G3, and -G4) and soluble forms (HLA-G5 and -G6) [20]. Whereas membrane HLA-G expression in trophoblast cells is restricted to extravillous trophoblast cells, which invade the maternal decidua, expression of soluble HLA-G was detected in all types of placental trophoblast cells [21]. To date, the precise role of HLA-G in the placenta remains unclear [22]. A common hypothesis proposes that expression of HLA-G on invading trophoblast cells is needed to prevent NK cell attack [9,23]. However, this might not be necessary as trophoblast cells are intrinsically resistant to NK cell–mediated lysis [24]. An alternative hypothesis proposes that trophoblast–NK cell interactions regulate expression of cytokines by NK cells to promote remodeling of the maternal vasculature, which is required to establish adequate blood supply to the fetus [5,25,26]. The benefit of NK cell activation during early pregnancy is supported by genetic studies on preeclampsia, a potentially fatal disease due to incomplete remodeling of spiral arteries by trophoblast cells. Resistance to preeclampsia correlated with combinations of fetal *HLA* genes and maternal *KIR* genes that seem to favor NK cell activation over NK cell inhibition [27].

In mice as well, NK cells are present in the uterine mucosa where they increase in number after implantation and become activated as decidualization occurs [28]. In both mice and humans, uterine NK cells have limited cytotoxic potential but produce cytokines including IFN-γ and tumor necrosis factor (TNF)-α [20,29]. In several different strains of genetically manipulated mice that lack NK cells, decidual integrity is compromised and spiral artery modification is minimal. In addition, IFN-γ produced by mouse uterine NK cells has been implicated in the remodeling of the vasculature of the maternal arteries necessary for a normal pregnancy [30]. The signals that induce activation of uterine NK cells are still unknown.

Results presented here support a role for KIR2DL4 in the activation of NK cells at sites of soluble HLA-G expression. Unlike other members of the KIR family, KIR2DL4 is localized predominantly in endosomes and mediates endocytosis of its ligand, soluble HLA-G. Furthermore, engagement of KIR2DL4 by soluble ligand activates a proinflammatory/proangiogenic response, consistent with a role in promoting vascularization during early pregnancy.

## Results

### Interferon-γ Secretion Is Induced by Soluble, but Not Plate-Coated, or Bead-Bound Antibodies to KIR2DL4

Resting NK cells typically express very low or undetectable levels of KIR2DL4 on the cell surface. Activation of NK cells with interleukin (IL)-2 and feeder cells resulted in a transient increase in the level of cell surface KIR2DL4 (Figure 1A). The increase usually peaked between 7 and 14 days, and by 3 weeks in culture, receptor levels were no longer detectable. However, despite low surface levels of KIR2DL4 on resting NK cells, secretion of IFN-γ was observed upon engagement by anti-KIR2DL4 mAbs [7]. Three different mAbs (IgG1 33 and IgM 36 and 64) in solution, and even purified Fab of mAb 33, could enhance IFN-γ secretion in the absence of cross-linking with secondary antibodies [7] (Figure 1B and unpublished data). In contrast, plate-coated or bead-bound mAb 33 did not enhance IFN-γ secretion (Figure 1B). The lack of stimulation by cross-linked antibodies was not due to induction of cell death, as determined with annexin-V–FITC and propidium iodide to detect apoptotic and necrotic cells (unpublished data). Activation by soluble antibodies to KIR2DL4 was in direct contrast to the response to other NK cell receptors such as CD16 or 2B4 (CD244), for which soluble antibodies were not sufficient to trigger a response, and cross-linking by plate-coating or by binding to beads was required to obtain comparable IFN-γ release (Figure 1B and unpublished data).

**Figure 1:**
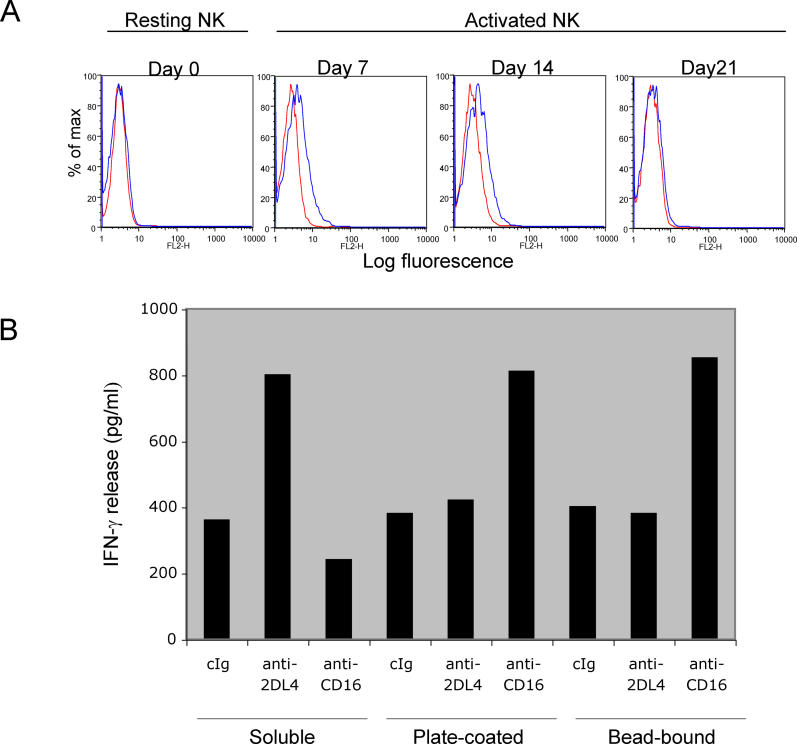
IFN-γ Secretion by Resting NK Cells Is Induced by Soluble Antibodies to KIR2DL4 (A) KIR2DL4 expression on the surface of NK cells immediately after isolation (day 0) or after 7, 14, or 21 d in culture with autologous feeder cells and rIL-2. Cells were stained with mAb 33. (B) Resting NK cells were incubated with soluble (10 μg/ml), plate-coated (5 μg/0.1 ml/well), or bead-bound (4 beads/cell) antibodies to KIR2DL4 (mAb 33) or CD16 (mAb 3G8). After 24 h, culture supernatants were tested by ELISA for IFN-γ production.

### Dynamin-Dependent Endocytosis of KIR2DL4

Activation by soluble antibodies to KIR2DL4, despite negligible receptor expression at the cell surface, could be explained if KIR2DL4 were actively endocytosed into resting NK cells. To examine the subcellular distribution of KIR2DL4, both resting and activated NK cells were fixed, permeabilized, stained with KIR2DL4-specific mAb 33, and analyzed by confocal microscopy. Most of the receptor resides in vesicular structures, in contrast to other KIR receptors such as KIR2DL1, which is detectable at the cell surface (Figure 2A). To test if active endocytosis was a mechanism for delivery of KIR2DL4 into intracellular compartments, resting NK cells were incubated with Cy3-conjugated mAb 33 at 37 °C for different times prior to analysis by confocal microscopy. As shown in Figure 2B, at 30 min, some of the KIR2DL4 had begun to internalize, and by 120 min, extensive endocytosis of KIR2DL4 was detected by the presence of the 33-Cy3 mAb in intracellular vesicles. The staining of internalized KIR2DL4 at 120 min was very similar to that detected by intracellular staining for total KIR2DL4 (Figure 2A). Endocytosis was also seen with the Fab portion of the KIR2DL4-specific mAb 33, ruling out Fc receptor–mediated internalization of mAb 33 (Figure 2B).

**Figure 2:**
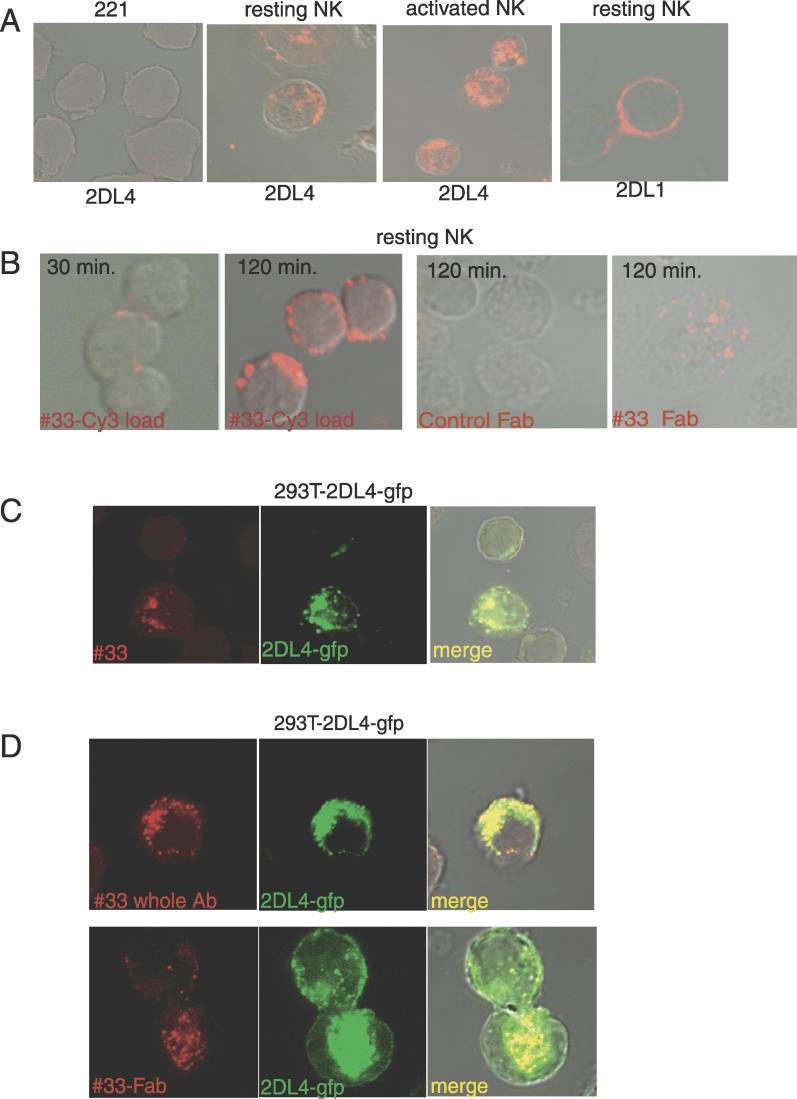
KIR2DL4 Is Endocytosed into Intracellular Vesicles (A) The B cell line 721.221 (221), resting NK cells, and activated NK cells were fixed, permeabilized, and stained with Cy3-conjugated mAb 33. In the image on the right, resting NK cells were fixed, permeabilized, and stained with a polyclonal antibody specific for the tail of KIR2DL1, followed by Alexa-568–conjugated secondary antibodies. (B) Resting NK cells were incubated at 37 °C for 30 min or 120 min with KIR2DL4-specific mAb 33-Cy3, with a Fab of control mAb 2A8 or with a Fab of anti-KIR2DL4 mAb 33, as indicated. Cells were then fixed and analyzed by confocal microscopy. (C) The 293T cells stably transfected with KIR2DL4-gfp (293T-2DL4-gfp) were fixed, permeabilized, and stained with anti-KIR2DL4 mAb 33 followed by Alexa-568–conjugated secondary antibodies to show co-localization with KIR2DL4-gfp. Single confocal sections are shown. (D) The 293T-2DL4-gfp cells were incubated at 37 °C for 120 min with anti-KIR2DL4 mAb 33, either intact (33 whole Ab) or as a Fab (33-Fab). Cells were fixed, stained with Alexa-568–conjugated secondary antibodies, and analyzed by confocal microscopy. Single confocal sections are shown.

An intracellular, vesicular pattern of KIR2DL4 distribution was also observed after stable transfection of gfp-tagged KIR2DL4 in the human embryonic kidney cell line 293T (Figure 2C, center). In addition, good co-localization of anti-2DL4 mAb 33 and gfp-tagged KIR2DL4 was observed, validating the specificity of the staining (Figure 2C). Uptake of mAb 33 was also detected in 293T-2DL4-gfp cells (Figure 2D). Endocytosis of KIR2DL4 was not induced by mAb-mediated cross-linking, because the purified Fab portion of mAb 33 was also endocytosed (Figure 2D). In both instances, mAb was internalized to the same compartments where endogenous KIR2DL4-gfp was localized (Figure 2D). These data show that KIR2DL4 can also be endocytosed in non-NK cells.

Next, we tested whether endocytosis of KIR2DL4 was dependent on the GTPase dynamin, a central player in clathrin-mediated endocytosis [31]. The endocytosis assay was performed in transfected 293T cells expressing HA-tagged KIR2DL4 and either wild-type gfp-tagged dynamin or a dominant-negative mutant (K44A) of gfp-tagged dynamin, which competes for dynamin function due to a defect in GTP binding and hydrolysis [32]. Cells were incubated with Cy3-conjugated anti-KIR2DL4 mAb 33 for 2 h and processed for confocal imaging. Cells expressing dynamin were identified by fluorescence of gfp. Whereas endocytosed KIR2DL4 could be seen in vesicular structures in the presence of wild-type dynamin, dynamin K44A hindered the transport of KIR2DL4 into vesicles and caused a striking redistribution of the receptor at the plasma membrane (Figure 3).

**Figure 3:**
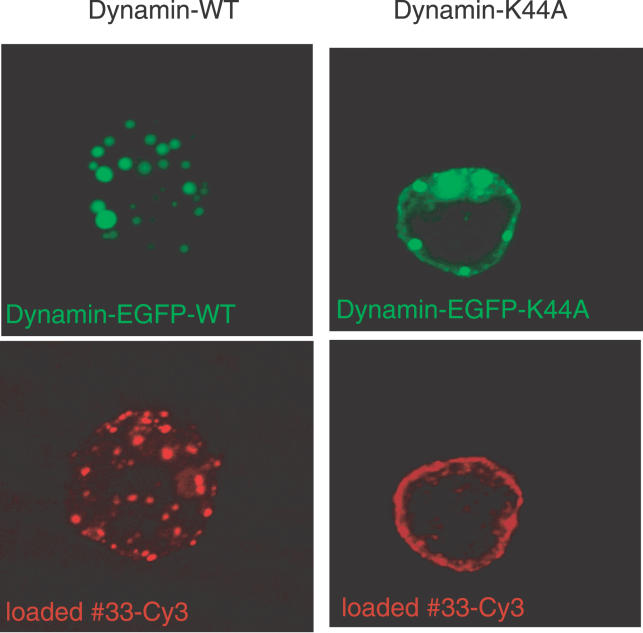
Internalization of KIR2DL4 Is Dynamin Dependent The 293T cells transfected with HA-tagged KIR2DL4 together with either wild-type dynamin-gfp (Dynamin Egfp-WT) or a dominant-negative mutant of dynamin (dynamin Egfp-K44A) were loaded with KIR2DL4-specific Cy3-conjugated mAb 33 for 120 min and fixed. Individual confocal sections are shown.

To visualize endocytic compartments containing KIR2DL4 at higher resolution, the NK cell line NKL and the NK cell line YTS stably transfected with KIR2DL4-gfp (YTS-2DL4-gfp) were loaded with mAb 33 for 120 min at 37 °C. Cells were fixed, incubated with horseradish peroxidase (HRP)-conjugated secondary antibodies, and reacted with 3,3′-diaminobenzidine substrate. Electron micrographs revealed HRP reaction product mainly in vesicles that ranged between 250 and 500 nm in size in both NKL and YTS-2DL4-gfp cells (Figure 4A). The majority of HRP-reactive vesicles contained a rim of HRP reaction product and an electron-lucent central region characteristic of early endosomes. Some of the vesicles also had more electron-dense content (Figure 4A). There was negligible vesicular staining of cells not loaded with mAb 33 (unpublished data). Staining of a few tubular structures and multivesicular bodies was also observed, consistent with localization in a subset of recycling endosomes and lysosomes, respectively (unpublished data). Thus, electron microscopy confirmed the vesicular localization of endocytosed KIR2DL4.

**Figure 4:**
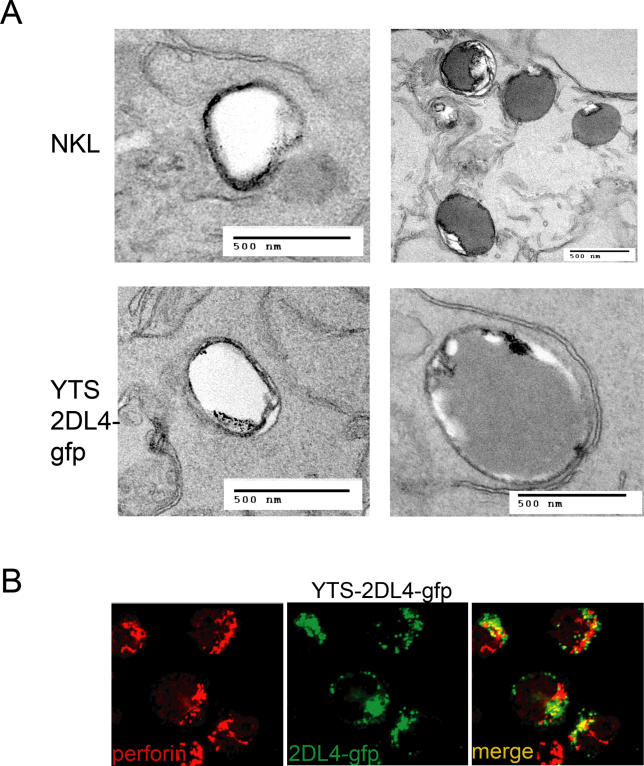
Immunolocalization of Endocytosed KIR2DL4 in NK Cells by Electron Microscopy (A) The NK cell lines NKL and YTS-2DL4-gfp were loaded with anti-KIR2DL4 mAb 33 for 120 min. Endocytosed receptor was detected using HRP-conjugated sheep anti-F(ab′)_2_ mouse IgG. The HRP reaction product visible as a dark stain identifies the location of endocytosed KIR2DL4. Vesicular structures positive for KIR2DL4 ranged between 250 and 500 nm in size. (B) The NK cell line YTS, stably transfected with KIR2DL4-gfp (YTS-2DL4-gfp), was fixed, permeabilized, and stained with antibody to perforin followed by Alexa-568–conjugated secondary antibodies. Single confocal sections are shown.

Confocal microscopy was performed with YTS-2DL4-gfp cells to test how KIR2DL4-containing vesicles relate to perforin-containing cytotoxic granules. Anti-perforin antibodies decorated vesicles in YTS-2DL4-gfp cells that were distinct from KIR2DL4-containing vesicles (Figure 4B). The lack of overlap was confirmed by Z-stack/three-dimensional reconstruction studies (unpublished data). Resting NK cells co-stained with mAb 33 and anti-perforin antibodies also revealed no overlap of KIR2DL4 with perforin (unpublished data). Lack of overlap was also seen with antibodies to granzyme A (unpublished data). The chemokine RANTES resides in specialized storage vesicles in T cells [33]. Confocal experiments revealed that RANTES was also in vesicular compartments in NK cells but that those compartments were distinct from vesicular compartments containing KIR2DL4 (unpublished data).

### KIR2DL4 Resides in Rab5^+^ Early Endosomes

Co-localization of KIR2DL4 with a number of markers for endocytic and lysosomal compartments was tested in resting NK cells and in 293T-2DL4-gfp cells. Rab5 and its effector EEA-1 are markers for early endosomes; mannose 6-phosphate receptor (M6PR) is a marker for late endosomes; and lysosome-associated membrane protein 1 (LAMP-1) is a marker for lysosomes. Resting NK cells were fixed, permeabilized, and stained with anti-KIR2DL4 mAb 33 and with Alexa-488–conjugated secondary antibodies, followed by staining with antibodies against Rab5, EEA-1, or M6PR and with Alexa-568–conjugated secondary antibodies (Figure 5 and unpublished data). The 293T-2DL4-gfp cells were stained for the same endocytic markers and KIR2DL4 was visualized by gfp fluorescence. In both resting NK cells and 293T-2DL4-gfp cells, significant overlap of staining was obtained only in the case of Rab5 (Figure 5). Even though Rab5 and EEA-1 identify early endosomes, the overlap of staining of KIR2DL4 with EEA-1 was more limited. This was confirmed by three-color confocal analysis to detect KIR2DL4, Rab5, and EEA-1 in the same cells (unpublished data). Likewise, there was limited overlap of KIR2DL4 staining with M6PR (Figure 5) and LAMP-1 (unpublished data).

**Figure 5:**
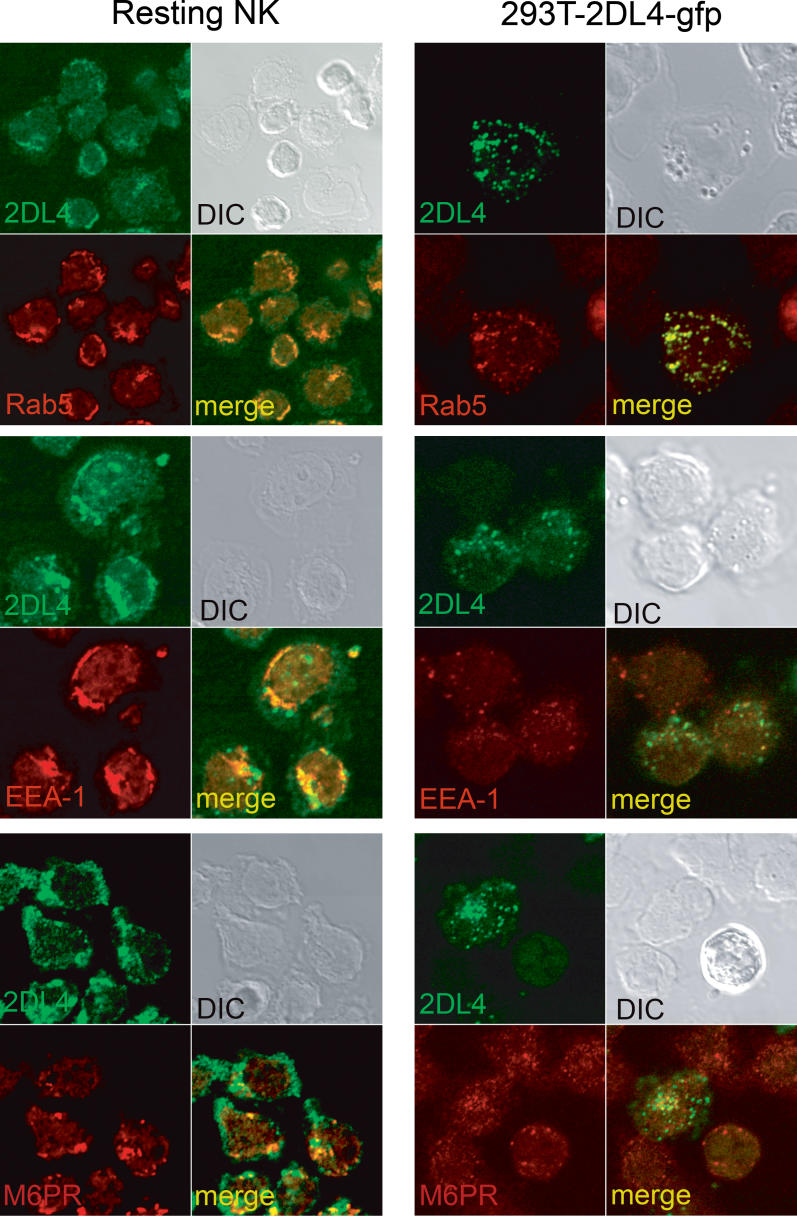
KIR2DL4 Resides in Endocytic Compartments Resting NK cells and 293T-2DL4-gfp cells were fixed, permeabilized, and stained with antibodies against Rab5, EEA-1, and M6PR, followed by Alexa-568–conjugated secondary antibodies. NK cells were further stained with mAb 33 coupled to Alexa-488 to detect KIR2DL4.

Members of the Rab GTPase family, such as Rab4, Rab5, Rab7, and Rab11, occupy distinct yet overlapping membrane domains on early and recycling endosomes and serve as determinants of organelle identity [34]. Rab5 is a key regulator of transport to early endosomes; Rab4 and Rab11 are implicated in recycling, from endosomes back to the plasma membrane; and Rab7 has a role in the late endocytic pathway and in lysosome biogenesis. To show unequivocally that KIR2DL4 co-localizes with Rab5 and to address the specificity of this co-localization, gfp-tagged versions Rab4, Rab5, Rab7, and Rab11 were co-expressed with HA-tagged KIR2DL4 in 293T cells. The cells were stained with anti-HA antibodies followed by Alexa-568–conjugated secondary antibodies, and confocal images were obtained. As seen previously with endogenous Rab5 (Figure 5), all KIR2DL4-containing vesicles corresponded to Rab5-positive compartments (Figure 6). In contrast, overlap with compartments containing other Rab proteins was minimal (Figure 6). Thus, KIR2DL4 resides in Rab5-containing subcompartments of the early endocytic pathway.

**Figure 6:**
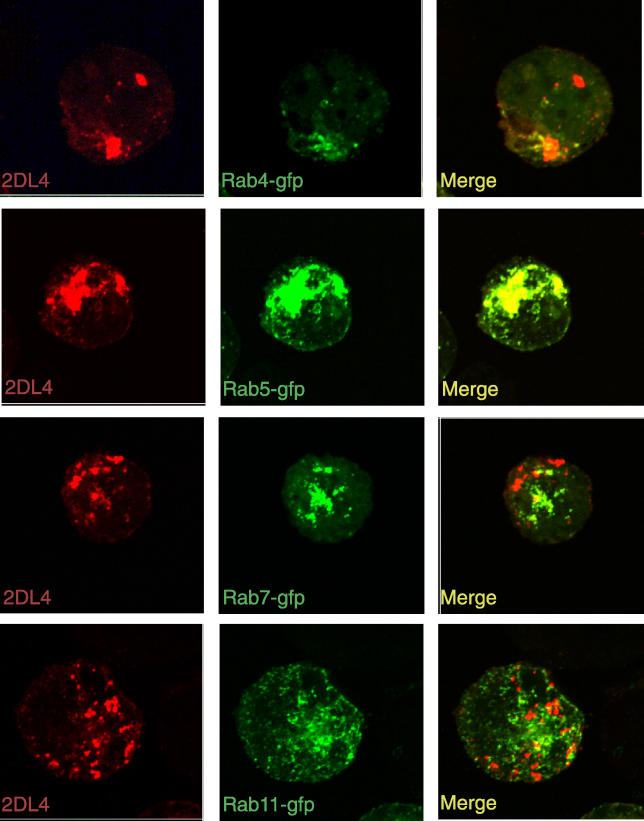
KIR2DL4 Localizes to a Subset of Endosomes Containing Rab5 The 293T cells were transfected with HA-tagged KIR2DL4 and gfp-tagged versions of Rab4, Rab5, Rab7, and Rab11. Forty hours after transfection, cells were fixed and stained with anti-HA mAb, followed by Alexa-568–conjugated secondary antibodies to detect KIR2DL4. Single confocal sections are shown.

### KIR2DL4 Binds Both Membrane and Soluble Forms of HLA-G

Soluble, recombinant Ig-fusion proteins of KIR2DL4 bound to cells expressing high levels of HLA-G [8,9]. However, one study could not reproduce these results [35], and soluble forms of HLA-G have not bound to NK cells [36] or to KIR2DL4 [35]. Failure to detect this interaction may reflect an intrinsic low affinity, as was previously noted for interactions between activating KIR and their HLA ligands [37,38]. The specificity of interaction between KIR2DL4 and HLA-G was explored further using mAbs to MHC class I and KIR2DL4 to block binding of KIR2DL4-Ig fusion proteins to cells expressing HLA-G. Background binding of KIR2DL4-Ig to 721.221 cells, which lack all classical HLA class I genes, was not inhibited by anti-class I mAbs but was reduced by anti-KIR2DL4 mAb 33 (Figure 7). In contrast, the enhanced binding of KIR2DL4-Ig to transfected 721.221 cells expressing membrane-bound HLA-G (221-G) was blocked by mAb DX17, a pan–class I antibody known to block KIR–HLA class I interactions [39], by the HLA-G–specific mAb G233, and by the KIR2DL4-specific mAb 33 (Figure 7). Thus, binding was blocked using specific mAbs to either KIR2DL4-Ig fusion protein or HLA-G expressed on 221-G cells.

**Figure 7:**
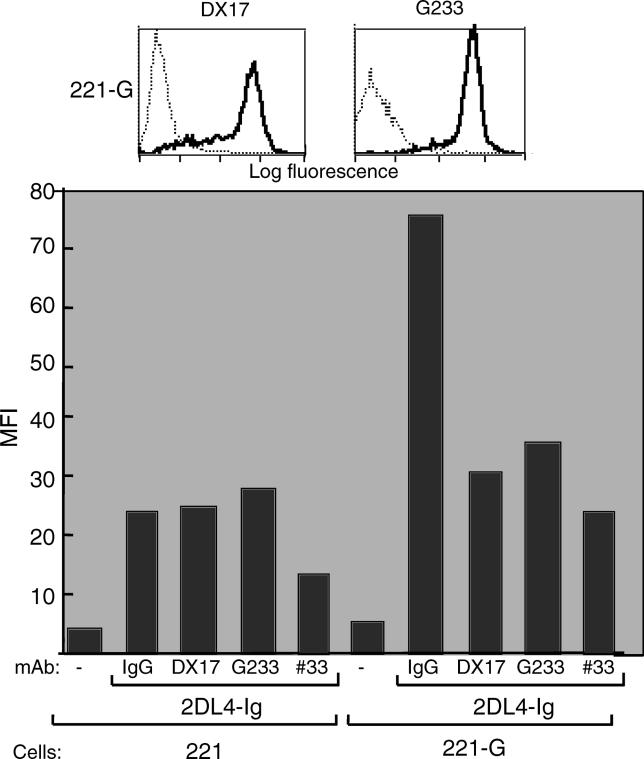
Binding of KIR2DL4-Ig Fusion Proteins to HLA-G–Expressing Cells Is Blocked by Anti-KIR2DL4 and Anti-HLA Class I mAbs (Top) 221-G cells were stained with mAb DX17 (pan–HLA class I mAb) and mAb G233 (HLA-G–specific mAb) (solid lines). Staining with secondary antibody alone is also shown (dotted lines). (Bottom) The 221 and 221-G cells were incubated with 50 μg/ml KIR2DL4-Ig fusion protein in the presence of 20 μg/ml of either isotype-matched control Abs or mAbs specific for class I (DX17), HLA-G (G233), or KIR2DL4 (33). Cells were then stained with goat anti-human IgG1 secondary antibodies and assessed by flow cytometry. The data are expressed as mean fluorescence intensity (MFI).

We explored the possibility that soluble HLA-G may be a natural ligand of KIR2DL4. To date, evidence that HLA-G binds directly to KIR2DL4 is lacking. Soluble HLA-G produced in *Escherichia coli* and refolded with β_2_-microglobulin and the peptide KGPPAALTL was tested for endocytosis into KIR2DL4-containing compartments. HLA-Cw3 produced in *E. coli* and refolded with β_2_-microglobulin and peptide GAVDPLLAL was used as a specificity control. This refolded HLA-C preparation bound to KIR2DL2 by surface plasmon-resonance analysis [40] (and P. Sun, personal communication). Staining of 721.221 cells expressing HLA-Cw3 with F4/326 is also included (Figure 8A) to show that the mAb can detect HLA-Cw3 in fixed and permeabilized samples. Resting NK cells were loaded with either refolded HLA-G or refolded HLA-Cw3 for 2 h. Internalized soluble HLA-G, but not HLA-Cw3, was detected in intracellular vesicles (Figure 8A). It was difficult to show unambiguous co-localization of KIR2DL4 and HLA-G in resting NK cells, possibly because HLA-G bound to KIR2DL4 interfered with the staining of KIR2DL4 by mAb 33. Therefore, co-localization experiments were done with the NK cell line YTS that was stably transfected with gfp-tagged KIR2DL4. Endocytosed HLA-G was detected in intracellular vesicles that contained gfp-tagged KIR2DL4 (Figure 8B). Extensive co-localization of refolded HLA-G and KIR2DL4-gfp was also seen after incubation for 2 h with 293T-2DL4-gfp cells (Figure 8C). As seen with resting NK cells, refolded HLA-Cw3 was not detected in compartments containing KIR2DL4-gfp in 293T-2DL4-gfp cells.

**Figure 8:**
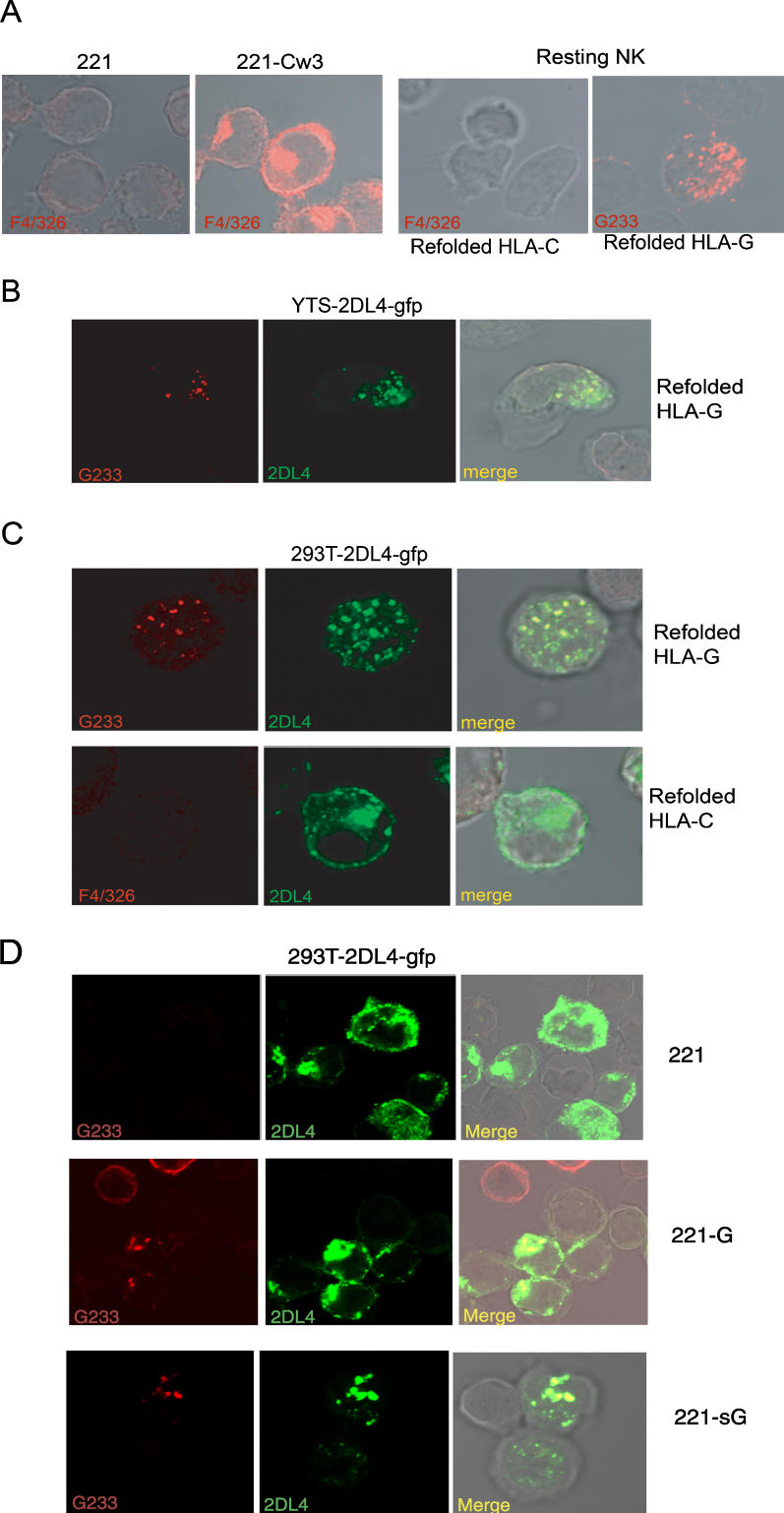
Cell Surface Shed and Secreted, Soluble HLA-G Is Endocytosed into KIR2DL4-Containing Vesicles (A) Endocytosis of soluble HLA-G in resting NK cells. The 221 cells and 221 cells transfected with HLA-Cw3 (221-Cw3) were fixed, permeabilized, and stained with mAb F4/326. Resting NK cells were incubated at 37 °C for 120 min with soluble, refolded HLA-C or HLA-G. Cells were then fixed, permeabilized, and stained with reagents to detect HLA-C (F4/326) or HLA-G (G233) as indicated. (B) The NK cell line YTS-2DL4-gfp was loaded at 37 °C for 120 min with refolded HLA-G. Cells were fixed, permeabilized, and stained with mAb G233 to detect co-localization of soluble HLA-G with gfp-tagged KIR2DL4. (C) Recombinant soluble molecules of HLA-G but not HLA-C are endocytosed into 293T-2DL4-gfp cells. Refolded HLA-G and HLA-C were incubated with 293T-2DL4-gfp cells for 2 h. Cells were then fixed, permeabilized, and stained with either mAb G233 (to detect endocytosed HLA-G; upper) or mAb F4/326 (to detect endocytosed HLA-C; middle). (D) The 293T-2DL4-gfp cells were co-cultured with an equal number of 221 cells, 221 cells expressing transmembrane HLA-G (221-G), and 221 cells expressing a soluble isoform of HLA-G (221-sG) for 48 h. Adherent 293T-2DL4-gfp cells were fixed, permeabilized, and stained with mAb G233 followed by Alexa-568–conjugated secondary antibodies prior to acquisition of confocal images. Two 221-G cells are visible in the middle panel.

Soluble HLA-G can be produced by alternative splicing [41] and from cell surface–expressed HLA-G by proteolytic cleavage [42]. Transfected 721.221 cells expressing membrane-bound HLA-G (221-G) or secreted HLA-G (221-sG) were mixed with untransfected 293T cells and with 293T cells stably expressing KIR2DL4-gfp. After 48 h of co-culture, cells were gently washed and the adherent 293T-2DL4-gfp cells were fixed, permeabilized, and stained with the HLA-G–specific mAb G233. No internalization of HLA-G occurred after co-culture with 221 cells (Figure 8A). In contrast, co-culture with either 221-G or 221-sG resulted in internalization of HLA-G in KIR2DL4-containing vesicles. Note the vesicular staining of endocytosed HLA-G in 293T-2DL4-gfp cells in contrast to the cell surface staining of HLA-G on the 221-G cells included for comparison (Figure 8A, middle row, left panel). No HLA-G was detected in untransfected 293T cells co-cultured with 221-G and 221-sG cells (unpublished data). Therefore, soluble HLA-G produced by shedding from the cell surface or by secretion of an alternatively spliced form is internalized by KIR2DL4 into endosomes.

To further quantify the interaction of KIR2DL4 with soluble HLA-G, a recombinant, single-chain HLA-G (sHLA-G) expressed in Chinese hamster ovary (CHO) cells was used. This single-chain HLA-G contains an HLA-G–binding peptide (RLPKDFVDL), the extracellular domains (α1, α2, α3) of HLA-G, and β_2_-microglobulin all connected via linkers [43]. 293T-2DL4-gfp cells were incubated for 2 h with sHLA-G at 37 °C. Cells were fixed, permeabilized, and stained with mAb G233. Vesicular staining of sHLA-G, which co-localized with gfp-tagged KIR2DL4, was visible, similar to that seen after incubation with soluble anti-2DL4 mAb 33 (Figure 9A). The fluorescence intensity of endocytosed sHLA-G was quantified and compared to that of KIR2DL4-gfp in 293T cells. The expectation was that sHLA-G uptake should increase with higher expression levels of KIR2DL4-gfp. Red fluorescence in vesicles was compared to the green fluorescence of KIR2DL4-gfp in 38 individual cells (Figure 9B). The correlation between the level of expression of gfp-tagged KIR2DL4 and the extent of sHLA-G loading was highly significant (*p* ≤ 0.0001).

**Figure 9:**
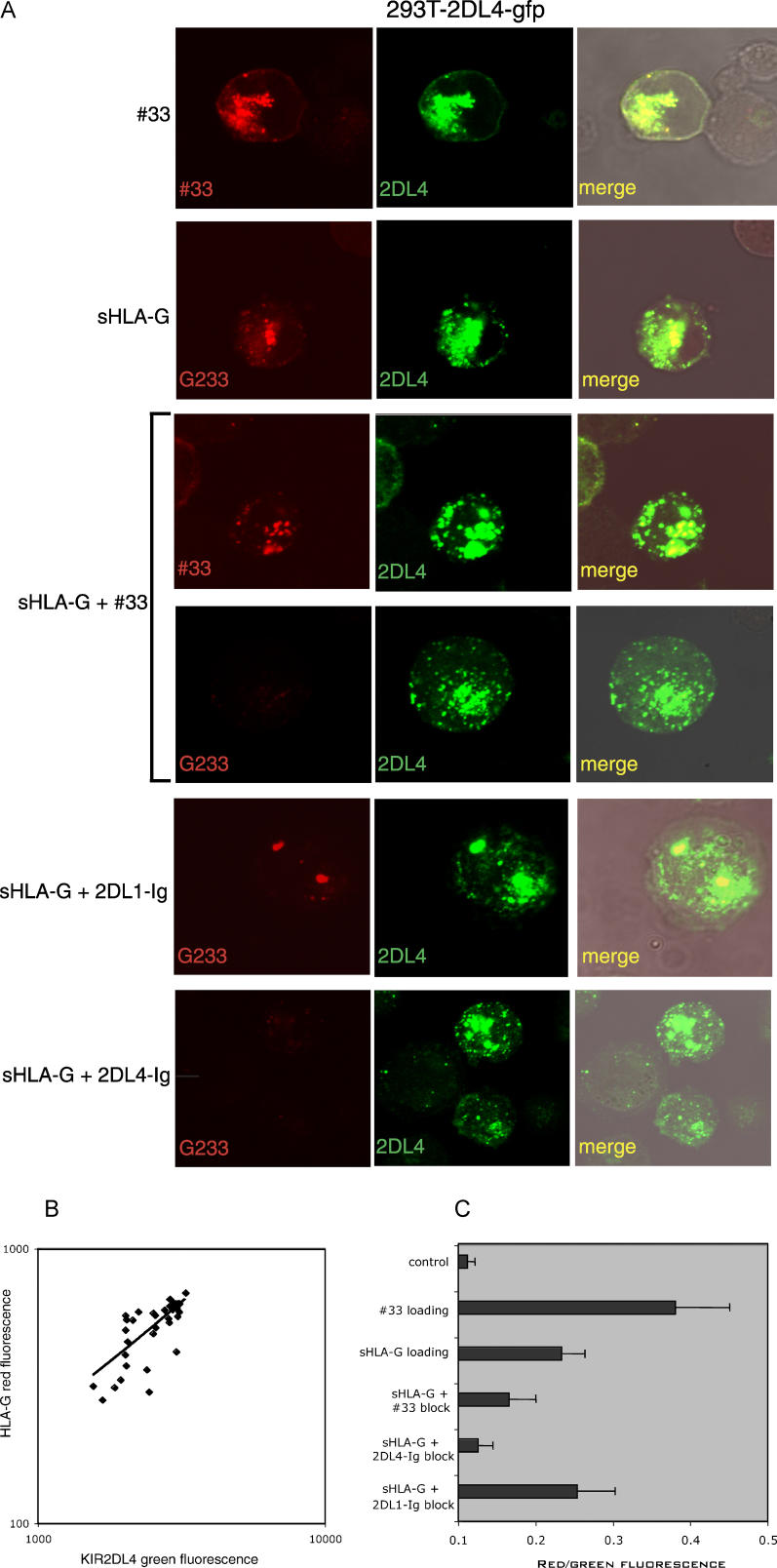
Endocytosis of Soluble HLA-G into 293T-2DL4-gfp Cells Is Blocked by Anti-KIR2DL4 mAb and by Soluble KIR2DL4 (A) Recombinant, soluble, sHLA-G at 50 μg/ml or mAb 33 (50 μg/ml) was incubated with 293T-2DL4-gfp cells. sHLA-G was also incubated together with 50 μg/ml mAb 33, 50 μg/ml KIR2DL1-Ig, or 50 μg/ml KIR2DL4-Ig, as indicated on the left. Cells were fixed, permeabilized, and stained with either Alexa-568–conjugated secondary antibodies to detect mAb 33 or anti-HLA-G mAb G233, as indicated. Individual confocal sections are shown. (B) Uptake of sHLA-G into 293T-2DL4-gfp cells correlates with level of KIR2DL4 expression. Red fluorescence intensity of G233 staining and green fluorescence intensity of gfp were quantified in 38 individual cells and plotted on a log scale. A best-fit line was generated by linear regression analysis using EXCEL data analysis software. (C) Ratio of red to green fluorescence was quantified for each loading condition as indicated. Average of 10 cells is shown, and standard deviation is shown as bars.

To test whether KIR2DL4 was involved in sHLA-G internalization, sHLA-G was incubated with 293T-2DL4-gfp cells in the presence of anti-KIR2DL4 mAb 33, which blocks KIR2DL4 binding to HLA-G on cells (Figure 7). Internalization of sHLA-G was blocked in the presence of mAb 33, whereas mAb 33 was still internalized in the presence of sHLA-G (Figure 9A and 9C). Similar results were obtained in YTS-2DL4-gfp cells (unpublished data). To test whether sHLA-G interacts directly with KIR2DL4, sHLA-G was loaded in the presence of soluble KIR2DL4-Ig fusion protein. Soluble KIR2DL1-Ig fusion protein with specificity for a subset of HLA-C alleles was used as a control. Soluble KIR2DL4-Ig prevented the uptake of sHLA-G into 293T-2DL4-gfp cells, whereas soluble KIR2DL1-Ig had no effect (Figure 9A and 9C). These results imply a direct interaction between soluble forms of HLA-G and KIR2DL4.

### Engagement of KIR2DL4 by Soluble Ligand Induces a Unique Profile of Cytokine/Chemokine Secretion

We had shown previously that resting NK cells could be stimulated with anti-KIR2DL4 mAbs to secrete IFN-γ [7]. To get further information on the transcriptional response activated by this receptor, exploratory DNA microarray experiments were performed. Freshly isolated resting NK cells were stimulated either with control, isotype-matched, soluble antibodies or with KIR2DL4–specific soluble IgM mAbs for 5 h. Among the approximately 14,000 genes examined, all the genes that were up-regulated greater than 2-fold in resting NK cells from at least two of three independent donors are listed in Figure 10A. Interestingly, only a small subset of genes showed up-regulation. They include an array of proinflammatory/proangiogenic cytokines, such as IL-6, IL-1β, TNF-α, and IL-23 (comprised of the IL-23α and IL-12β subunits), and chemokines, such as IL-8, MIP-3α, MIP-1δ, MIP-1α, and MIP-2β.

**Figure 10:**
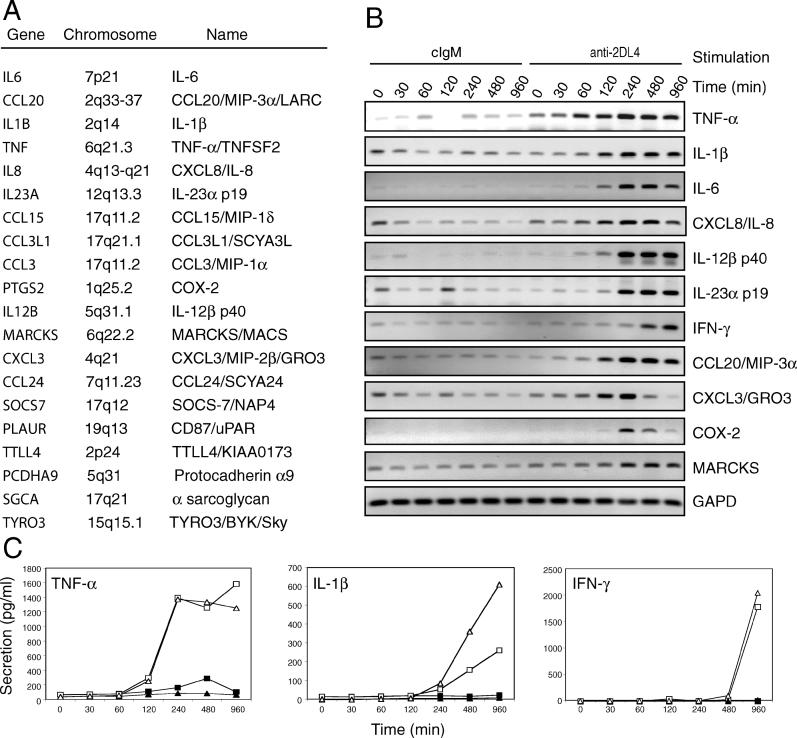
Binding of Soluble mAb to KIR2DL4 Up-regulates Multiple Genes in Resting NK Cells (A) All genes exhibiting greater than 2-fold up-regulation in microarray experiments with resting NK cells of at least two of three different individuals are listed. (B) Semiquantitative RT-PCR was performed on total RNA isolated at different time points from resting NK cells stimulated with either control IgM mAbs or anti-KIR2DL4 IgM mAbs 36 and 64. (C) Time course of TNF-α, IL-1β, and IFN-γ secretion by resting NK cells stimulated with anti-KIR2DL4 mAbs 36 and 64 (open symbols) or control IgM mAbs (closed symbols). Squares and triangles represent data obtained from two different donors. Protein secretion was detected by ELISA.

The KIR2DL4-induced expression of the majority of these genes was confirmed by semiquantitative RT-PCR analysis of RNA isolated at different timepoints after stimulation of resting NK cells by either control or anti-KIR2DL4 mAbs (Figure 10B). Such a time course analysis highlighted differences in the kinetics of responses. Transcription of the GRO3 and COX-2 genes was up-regulated transiently, whereas all of the other genes examined showed sustained expression up to the 16 h timepoint. Responses detectable within the first two hours included TNF-α, IL-1β, IL-8, MIP-3α, and GRO-3. In contrast, transcription of IL-6, IL-12β(p40), IL-23α(p19), COX-2, and MARCKS increased only after 2 h. Previous work [7] and Figure 1B show that stimulation by KIR2DL4 induces IFN-γ production. However, *IFNG* gene transcription was up-regulated only approximately 1.5-fold in the microarray studies and therefore did not score as positive. RT-PCR analysis reconciled this apparent discrepancy, as induction of *IFNG* transcription occurred only 8 h after stimulation. The time course of IFN-γ protein secretion supported the transcriptional data (Figure 10C). There is delayed secretion of IFN-γ compared to other cytokines such as TNF-α and IL-1β. *IFNG* gene transcription is a late response, which requires de novo protein synthesis, as it was inhibited by cycloheximide (Y. T. Bryceson, unpublished observation). Thus, exploratory microarray analysis using anti-KIR2DL4 mAbs to stimulate resting NK cells revealed the up-regulation of a select number of proinflammatory/proangiogenic cytokine and chemokine genes. However, it is possible that stimulation of KIR2DL4 by a natural ligand, such as soluble HLA-G, induces a different response.

To test whether a natural ligand of KIR2DL4 could confirm the observed transcriptional responses induced by anti-KIR2DL4 mAbs, protein expression was tested after incubation of resting NK cells with soluble HLA-G. Resting NK cells were incubated with sHLA-G produced in mammalian cells and tested for secretion of a number of cytokines and chemokines. As specificity control for activation by soluble HLA-G, mAb G233, which blocks the interaction between HLA-G and KIR2DL4 (Figure 7), was used. As shown in Figure 11, soluble KIR2DL4-specific mAb and sHLA-G activated the secretion of cytokines and chemokines, for which transcriptional activation had been observed in DNA microarrays. Responses induced by sHLA-G were inhibited by anti–HLA-G mAb G233 but not by an isotype-matched control IgG. Among the three donors tested, quantitative variation in protein secretion was observed. To take such variability into account, the amount of cytokine/chemokine induced by sHLA-G was compared to that induced by anti-KIR2DL4 mAb for each donor separately. For six of eight cytokines/chemokines tested, sHLA-G induced at least 50% of the amount induced by anti-KIR2DL4 mAb (Figure 11). To further evaluate the specificity of responses, secretion of IL-5, IL-12 (which shares the IL-12β p40 subunit with IL-23), and IL-13 was also determined. NK cells can produce IL-5 and IL-13 under certain conditions but have not been reported to produce IL-12. No secretion of any of these three cytokines was induced by anti-KIR2DL4 and by sHLA-G (unpublished data). Therefore, soluble HLA-G activates a qualitatively and quantitatively similar cytokine/chemokine response as KIR2DL4-specific mAb.

**Figure 11:**
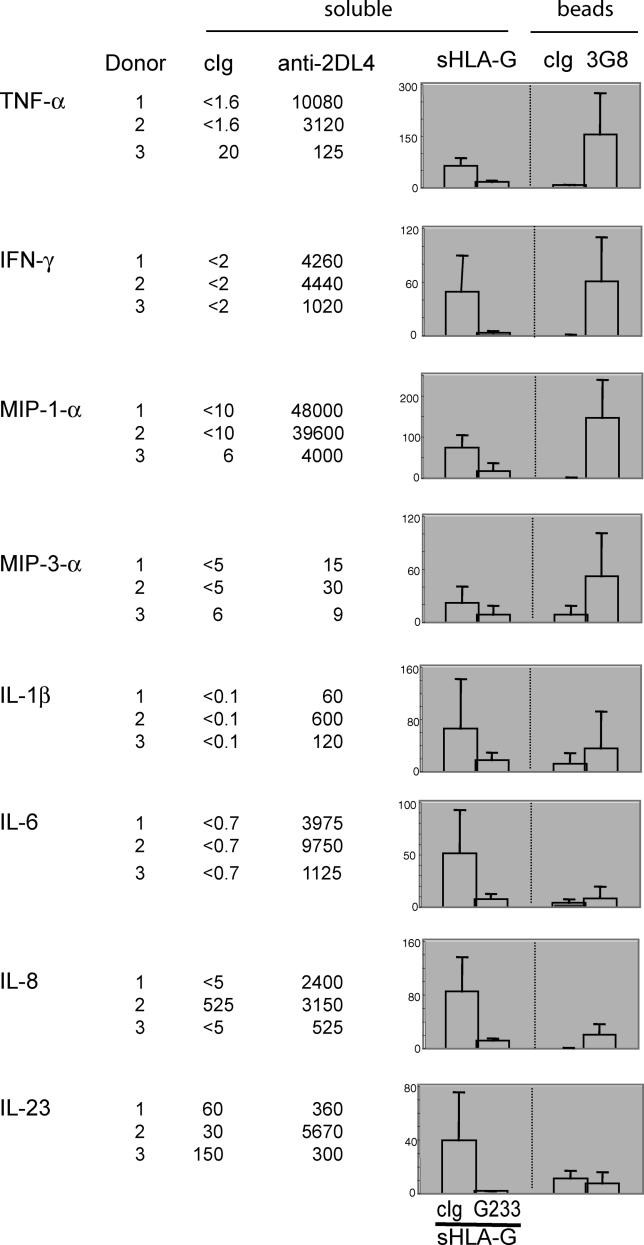
Cytokine/Chemokine Synthesis Induced by Soluble HLA-G in Resting NK Cells Resting NK cells (5 × 10^5^ cells/well) from three different donors were incubated separately for 48 h with either soluble, control IgM Ab (cIg), soluble anti-KIR2DL4 IgM mAb 36 (anti-2DL4), soluble HLA-G produced in CHO cells (sHLA-G), or beads coated with control anti-HA IgG1 mAb 16B12 (cIg) or with anti-CD16 IgG1 mAb 3G8 (3G8), as indicated. sHLA-G was used together with control IgG2a mAb (cIg) or with anti-HLA-G IgG2a mAb G233 (G233), as indicated. Secretion of the cytokines/chemokines listed on the left is given in pg/ml for each donor separately. Secretion induced by sHLA-G and by Ab-coated beads was compared to that induced by anti-KIR2DL4 mAb for each donor separately and is expressed as a percentage of the anti-KIR2DL4 response. The graphs represent the average ± standard deviation from three experiments.

To test whether the KIR2DL4-induced response was unique, responses elicited by CD16 cross-linking were evaluated in parallel. Anti-CD16 mAb attached to beads induced secretion of only a subset of the cytokines/chemokines induced by KIR2DL4 engagement (Figure 11). Very little or no secretion of IL-6, IL-8, and IL-23 was induced by CD16 cross-linking. Therefore, activation of KIR2DL4 by soluble ligand induces secretion of a unique profile of proinflammatory/proangiogenic mediators, which may be relevant to functional interaction between KIR2DL4 on uterine NK cells and HLA-G produced by trophoblast cells.

### KIR2DL4 Induces IL-8 Secretion in 293T Cells

The 293T cells are capable of producing IL-8, as TLR-4 agonists induced IL-8 secretion in TLR-4–transfected 293T cells [44]. As KIR2DL4 engagement in resting NK cells resulted in secretion of IL-8, the ability of KIR2DL4 to induce IL-8 secretion in 293T cells was tested. Transfected 293T cells expressing receptors 2B4 and gp49B were used for comparison. Expression of KIR2DL4, but not 2B4 or gp49B, in 293T cells induced IL-8 secretion (Figure 12A). As 293T cells did not stain with anti-HLA-G mAb G233 (see Figure 8D and unpublished data), IL-8 secretion may be induced by KIR2DL4 expression alone, independent of ligand binding. IL-8 expression in KIR2DL4-transfected 293T cells was used as a convenient assay to test mutants of KIR2DL4 (Figure 12B) for their ability to signal in 293T cells. A KIR2DL4 molecule lacking the arginine residue in the transmembrane region [2DL4(RY-GT)] was tested. This arginine may contribute to KIR2DL4 pairing with the FcRI γ chain [15], similar to other activating receptors that carry a charge in the transmembrane region. Second, a truncated KIR2DL4 (2DL4-TR), lacking its cytoplasmic tail, was tested. Confocal microscopy showed that both of these mutants were targeted to endocytic vesicles in 293T cells, similar to wild-type KIR2DL4 (unpublished data), showing that the cytoplasmic tail of KIR2DL4 is not required for endosomal targeting. Secretion of IL-8 was obtained with the 2DL4(RY-GT) mutant (Figure 12C), showing that the charged amino acid in the transmembrane region is not required for signaling. In contrast, the 2DL4-TR mutant did not signal for IL-8 secretion (Figure 12C), despite its localization in endosomes, pointing to a role for the transmembrane region and/or cytoplasmic tail in signaling. These results suggested that the natural *9A* allele of KIR2DL4, which encodes a receptor with a truncated cytoplasmic tail, would not signal for IL-8 secretion. A frameshift mutation was introduced in wild-type KIR2DL4 cDNA in order to produce a molecule identical to the KIR2DL4 9A allotype: consistent with the truncated mutant, expression of the 9A mutant in 293T cells did not induce IL-8 secretion (unpublished data).

**Figure 12:**
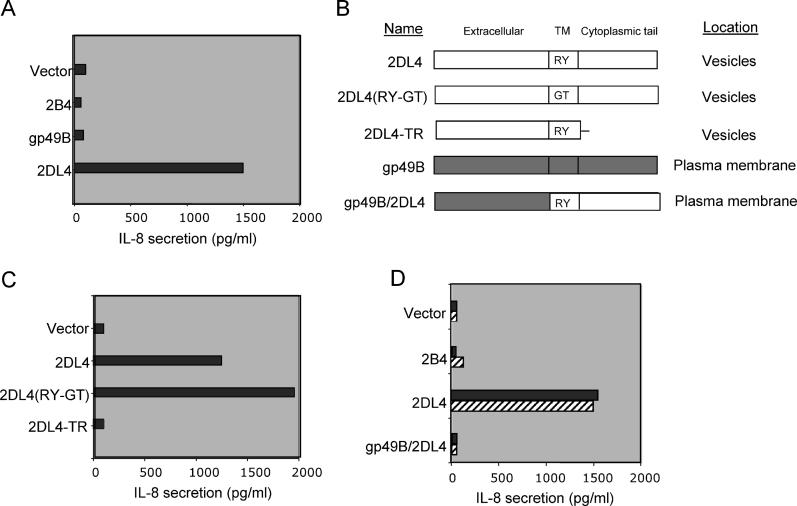
IL-8 Secretion Induced by KIR2DL4 Is Independent of the Transmembrane Arginine Residue and Requires Trafficking to Intracellular Vesicles (A) Expression of KIR2DL4 in 293T cells induces IL-8 secretion. The 293T cells were transfected with HA-tagged 2B4, gp49B, and KIR2DL4. After 48 h, culture supernatants were tested for IL-8 by ELISA. (B) Schematic representation of KIR2DL4 variants used in this study. Receptor localization was determined by confocal analysis of immunofluorescence staining of 293T cells transfected with the indicated constructs for 48 h. (C) IL-8 secretion induced by KIR2DL4 mutants in 293T cells. The 293T cells were transfected with HA-tagged KIR2DL4, KIR2DL4(RY-GT), and KIR2DL4-TR. After 48 h, culture supernatants were tested for IL-8 by ELISA. Transfection efficiency was verified by monitoring HA-positive cells by confocal microscopy. (D) Cell surface–expressed gp49B/2DL4 chimera does not induce IL-8 secretion. The 293T cells were transfected with HA-tagged 2B4, KIR2DL4, and gp49B/2DL4 chimera, as indicated. After 48 h, beads coated with control IgG (solid bars) or anti-HA mAb (hatched bars) were added at four beads per cell. Twelve hours later, culture supernatants were tested for IL-8 secretion. The percentage of 293T cells expressing receptor at the cell surface was monitored by HA staining and flow cytometry, and was as follows: vector control, 2%; 2B4, 39%; KIR2DL4, 9%; gp49B, 31%; gp49B/2DL4, 26%.

Another mutant was informative with respect to the signaling function of KIR2DL4. A chimeric, HA-tagged receptor containing the extracellular regions of gp49B fused to the transmembrane and cytoplasmic domains of KIR2DL4 was expressed at the surface of 293T cells and did not traffic to endosomes (Figure 12B), indicating that the luminal domain of KIR2DL4 is required for proper targeting. No IL-8 secretion was induced by this chimeric gp49B/2DL4, even after stimulation for 12 h with beads coated with anti-HA antibodies (Figure 12D). Cross-linking HA-tagged 2B4 with the same beads on 2B4-transfected 293T cells resulted in a very modest increase in IL-8 secretion. These results suggest that endosomal localization of KIR2DL4 is required for its signaling function.

## Discussion

The NK cell receptor KIR2DL4 emerges as an activation receptor specific for soluble HLA-G. Constitutive endocytosis of KIR2DL4 occurs in freshly isolated, resting NK cells, in IL-2–activated NK cells, in NK cell lines, and in 293T cells transfected with KIR2DL4. At steady state, most of KIR2DL4 is intracellular, which explains the difficulty in detecting cell surface receptor by flow cytometry. Endocytosis explains also the unusual property of KIR2DL4 to induce cytokine and chemokine secretion in response to soluble mAbs and to soluble HLA-G, in the absence of further cross-linking, but not in response to solid-phase mAbs (either plate-coated or bead-bound). Therefore, lack of detectable KIR2DL4 at the cell surface should not be taken as evidence for a lack of function. Endocytosed KIR2DL4 showed extensive co-localization with Rab5, a GTPase that marks a subset of early endosomes.

Endosomes have emerged recently as a specialized signaling compartment [45,46]. Some endocytosed receptors, such as the epidermal growth factor receptor and Toll-like receptor 9, signal from endosomes [45–47]. It is thought that endocytic vesicles carry signaling platforms where endocytosed receptors link with downstream signaling molecules and provide sustained signaling [45]. Our data strongly support signal transduction by KIR2DL4 in endosomes. First, KIR2DL4 is actively internalized into endosomes, where most of KIR2DL4 resides at steady state. Second, solid-phase Abs to KIR2DL4 did not induce IFN-γ secretion, indicating that cross-linking KIR2DL4 at the cell surface does not result in signaling. Third, expression of KIR2DL4 and of KIR2DL4 mutants in 293T cells resulted in secretion of IL-8 only for those receptors that were targeted to endosomes. A chimeric receptor with the transmembrane region and cytoplasmic tail of KIR2DL4 that remained at the cell surface did not activate IL-8 secretion, even after cross-linking at the cell surface. Extensive co-localization of endocytosed KIR2DL4 with a subset of early endosomes that carry the small GTPase Rab5 is interesting, as Rab5 was recently assigned a role in endosomal signaling by the epidermal growth factor receptor and by Toll-like receptor 9 [45,46]. The signaling pathway triggered by KIR2DL4 requires further study. Ligand-bound KIR2DL4 must somehow be distinguished from free, endogenous KIR2DL4 in NK cells, as resting NK cells do not respond to KIR2DL4 until soluble ligand is added. This distinction may not be made in transfected 293T cells, which appear to respond to KIR2DL4 in the absence of a ligand. Thus, KIR2DL4 signaling is subject to tight regulation in NK cells.

Signaling by KIR2DL4 in 293T cells also implies that immunoreceptor tyrosine-based activation motif (ITAM)-containing subunits, such as the FcRI γ chain, are unlikely to be necessary. This conclusion is further supported by the lack of requirement for the arginine residue in the transmembrane region of KIR2DL4 for signaling in 293T cells. Unlike other NK cell activation receptors with charged residues in the transmembrane region, such as NKG2D and NKp46, which require a partner chain for transport out of the endoplasmic reticulum, KIR2DL4 is readily transported to the cell surface and to endosomes, even in the absence of a known adapter. The position of the arginine at the fourth amino acid position from the N-terminus of the transmembrane region, rather than deeper into the lipid bilayer, suggests that membrane insertion may not be compromised [48]. However, induction of cytotoxicity by IL-2–activated NK cells through KIR2DL4 in a redirected lysis assay had been dependent on the arginine-tyrosine motif in the transmembrane region of KIR2DL4 [8]. It is therefore likely that KIR2DL4 can signal in different ways, for cytokine secretion independent of an arginine-mediated association (this study), and for cytotoxicity through an arginine-mediated association with the FcRI γ chain [8,15]. The biological context in which KIR2DL4-mediated cytotoxicity would occur is not clear. On the other hand, stimulation of a proinflammatory or proangiogenic response by soluble HLA-G could be a useful property in various physiological contexts. In particular, our study supports a new model for the role of NK cells in early pregnancy.

A ligand of KIR2DL4 is the nonclassical MHC class I molecule HLA-G, which is produced naturally in soluble form by alternative splicing [41,49] and by proteolytic cleavage of membrane-bound HLA-G [42]. In vitro studies have shown that HLA-G induces apoptosis of T cells, inhibition of alloproliferation of T cells and unfractionated mononuclear cells, and inhibition of NK cell cytotoxicity [19,42,50,51]. In contrast, HLA-G stimulated proliferation and cytokine secretion in uterine NK cells [29]. In this study, four different soluble forms of HLA-G have been internalized into KIR2DL4-containing endosomes. HLA-G shed from the cell membrane and the naturally secreted form of HLA-G, each produced in transfected 721.221 cells, were endocytosed into KIR2DL4-containing compartments of co-cultured cells. In addition, recombinant HLA-G, produced in *E. coli* and refolded with β2-microglobulin and peptide, was also internalized, as was an sHLA-G produced in transfected CHO cells. Competition for HLA-G uptake with a soluble, recombinant KIR2DL4-Ig fusion protein established, for the first time, a direct interaction between KIR2DL4 and HLA-G. So far, evidence for KIR2DL4 binding to HLA-G had been indirect. Binding of soluble KIR2DL4-Ig fusion proteins to cells expressing high levels of HLA-G [8,9] had been detected, but binding of soluble HLA-G tetramers to NK cells or soluble HLA-G to KIR2DL4 in surface plasmon resonance studies had not been detected [35,36]. The detection of KIR2DL4–HLA-G interaction may have been possible here due to accumulation of soluble HLA-G into KIR2DL4-containing endosomes.

In pregnancy, tolerance of the semiallogeneic fetus by the maternal immune system has long presented an immunological paradox [52,53]. To avoid rejection of the fetus, several factors must regulate maternal responsiveness to fetal alloantigens. Regulatory mechanisms may include inhibitory receptors on lymphocytes that bind ligands on fetal cells [5,54] and suppression of T lymphocytes by tryptophan catabolism via indoleamine-2,3-dioxygenase activity [55]. An alternate and nonexclusive view is that interactions of fetal cells with immune cells at the maternal–fetal interface play a role in proper implantation by shaping vascular adaptations of the surrounding maternal tissue via cytokine secretion [25,56]. Uterine tissue in early human pregnancy is characterized by extensive vascular remodeling, by invasion of HLA-G–expressing trophoblast cells, and by an abundance of maternal NK cells. Engagement of KIR2DL4 by soluble ligand induced a profile of gene transcription and protein secretion that is consistent with a proangiogenic response. The controversial role of HLA-G expression on extravillous cytotrophoblast cells—be it for inhibition or activation of maternal NK cells—may be settled in favor of activation of NK cells through soluble HLA-G for the purpose of vascular remodeling. Successful implantation and placentation require vascular endothelial growth factor, an angiogenic growth factor that is secreted by trophoblast cells throughout gestation [57]. The products of several genes up-regulated on NK cells by KIR2DL4 engagement, including TNF-α, IL-1β, and IFN-γ, induce vascular endothelial growth factor production in trophoblast cells, thereby influencing uterine angiogenesis [58]. Another gene induced by KIR2DL4 in NK cells, COX-2, is a rate-limiting enzyme in prostaglandin biosynthesis, which is required for uterine angiogenesis during implantation in mice [59]. In addition, IL-8 is known to induce neovascularization in tumor and viral models [60,61]. Secretion of IL-23, which is encoded by *IL12B* and *IL23A* genes, was also up-regulated by KIR2DL4 engagement with soluble ligands. Selective induction of these two genes occurs in mouse uterine NK cells at gestation day 6 [62]. In summary, the spectrum of proangiogenic mediators that are induced in resting NK cells by soluble HLA-G and by anti-KIR2DL4 mAb supports a role for KIR2DL4 in facilitating implantation and neovascularization during pregnancy. Our data, together with evidence that secretion of soluble HLA-G by in vitro fertilized human embryos is associated with a higher pregnancy rate [63,64] and that reduced levels of soluble HLA-G during early pregnancy correlate with a higher incidence of preeclampsia [65], suggest that KIR2DL4 has a positive role in reproductive success.

The description of soluble HLA-G expression in other cell types and tissues, such as erythroblasts during erythropoiesis [66], suggests the interesting possibility that a KIR2DL4-mediated signal in NK cells could serve a proinflammatory or proangiogenic function in other contexts as well. In this study, we show that constitutive internalization of KIR2DL4 results in uptake of soluble HLA-G and accumulation into endocytic compartments where the bulk of KIR2DL4 resides. Binding of soluble agonist by KIR2DL4 has the potential advantage of promoting specific NK cell responses without the complicating contribution of multiple receptor–ligand interactions that occur during cell–cell contacts, which engage a wide array of activating and inhibitory NK cell receptors.

## Materials and Methods

### Cell lines and culture

The 293T cells were obtained from ATCC (American Type Culture Collection, Manassas, Virginia, United States); YTS cells expressing an ecotropic receptor were a gift from G. Cohen (Harvard Medical School, Boston, Massachusetts, United States); NKL cells were a gift from M. Robertson (Indiana University School of Medicine, Indianapolis, Indiana, United States); and 221-HLA-Cw3 cells were a gift from J. Gumperz and P. Parham (Stanford University, Palo Alto, California, United States). The 221-HLA-G cells that do not express HLA-E [54] (referred to here as 221-G) cells were a gift from M. Lopez-Botet (University Pompeu Fabra, Barcelona, Spain); 221 cells transfected with the soluble form of HLA-G (221-sG) have been described previously [67]. NKL cells were cultured in RPMI 1640 medium containing 10% fetal calf serum, 1% l-glutamine, 1% sodium pyruvate, and 200 U/ml of recombinant IL-2 (National Cancer Institute–FCRDC, Frederick, Maryland, United States). All other cell lines were cultured in Iscove's medium supplemented with 10% fetal calf serum and 1% l-glutamine.

Human polyclonal NK cells were isolated from peripheral blood lymphocytes (PBL) using the MACS NK cell negative isolation kit (Miltenyi Biotech, Auburn, California, United States). NK cells were greater than 95% CD3− and CD56+. These freshly isolated NK cells (“resting NK”) were cultured in Iscove's medium containing 10% human serum without added IL-2 or feeders. “Activated NK” refers to NK cell populations from a given donor that was expanded with autologous irradiated PBL as feeders and IL-2, as described previously [7].

The 293T cells were transiently transfected with LipofectAMINE 2000 according to the supplier's instructions. The 293T-2DL4-gfp cells are 293T cells that were stably transfected, using LipofectAMINE 2000, with a KIR2DL4 cDNA fused to gfp in the vector pBABE (puro)CMV^+^ using SalI and BamH1 sites. Transfected cells were selected and maintained in Iscove's medium containing puromycin at a concentration of 2 μg/ml. YTS-2DL4-gfp refers to YTS cells transduced as described [68] to stably express the KIR2DL4-gfp fusion protein.

### Antibodies and reagents

mAbs to KIR2DL4, namely, 33 (IgG1), 36, and 64 (IgM), were generated in our laboratory [7]. To prepare Fab fragments, the 33 antibody was produced in mouse ascites fluid and the IgG1 was precipitated with 40% saturated ammonium sulfate and purified from the precipitate by ion-exchange chromatography on DEAE-Sepharose using an NaCl gradient. The IgG1 was then cleaved with papain at an enzyme-to-antibody ratio (wt/wt) of 1:20 in 100 mM Tris-HCl, 150 mM NaCl, 2 mM mercaptoethanol, 2 mM NaEDTA (pH 7.1) at 37 °C for 5 h. The reaction was quenched by addition of fresh iodoacetamide to a final concentration of 7.5 mM. Fab was purified from the digestion mixture by DEAE-Sepharose chromatography. The Fab fractions were pooled and concentrated in 20 mM Tris-HCl (pH 7.4). The integrity and purity of the Fab fragments were confirmed by SDS-PAGE analysis. The control Fab portion of IgG1 mAb 2A8, specific for malaria protein PV525, was obtained from K. Singh (National Institute of Allergy and Infectious Diseases [NIAID], National Institutes of Health [NIH], Rockville, Maryland, United States).

Antibodies to CD16 (3G8), Rab5 (610457), and EEA-1 (BD61045) were purchased from Becton Dickinson (Franklin Lakes, New Jersey, United States) Antibody to M6PR was purchased from Affinity Bioreagents (Golden, Colorado, United States), and antibody to perforin was purchased from Endogen Pierce (Rockford, Illinois, United States). An affinity-purified polyclonal rabbit antibody specific for the carboxy-terminal end of KIR2DL1 has been described [69]. MAb DX17 specific for MHC class I was a gift from L. Lanier (University of California San Francisco, California, United States), and mAb G233 specific for HLA-G was a gift from A. Moffett (Cambridge University, Cambridge, United Kingdom). MAb F4/326, specific for HLA-C alleles, was a gift from S.-Y. Yang (New York University, New York, New York, United States). Anti-HA mAb was obtained from Cell Signaling (Beverly, Massachusetts, United States), and all isotype control mAbs were obtained from Sigma (St. Louis, Missouri, United States). All secondary goat anti-mouse antibodies conjugated to either Alexa-Fluor 488 or Alexa-Fluor 564 were obtained from Molecular Probes (Eugene, Oregon, United States).

Rab5-gfp, Dynamin Egfp-WT, and Dynamin Egfp-K44A mutant constructs were obtained from J. Bonifacino (National Institute of Child Health and Human Development, NIH). Constructs encoding Rab4-gfp, Rab7-gfp, and Rab11-gfp cloned in pEgfp-C and C3 vectors (Clontech, Palo Alto, California, United States) were obtained from M. Zerial (Max Planck Institute for Molecular Cell Biology and Genetics, Dresden, Germany). These constructs have been previously described [70]. Expression constructs for transfection into 293T cells consisted of cDNAs for 2B4 [71], mouse gp49B [72], KIR2DL4, and KIR2DL4 variants cloned into the pDisplay vector (Stratagene, La Jolla, California, United States) that introduces an HA-tag at the amino-terminus. The KIR2DL4(RY-GT) construct was generated by site-directed mutagenesis of R246G and Y247T. The truncated KIR2DL4-TR was engineered by introduction of a stop codon at position K269 and contains the amino acids CSKK at the carboxy-terminus. The gp49B/2DL4 chimera was engineered using PCR to fuse the extracellular domain of gp49B ending at S226 with the transmembrane and tail of KIR2DL4 beginning at position S224. The amino acids at the boundary are DQSS/SWPS.

KIR2DL1-Ig and KIR2DL4-Ig fusion proteins have been described previously [8]. The recombinant, single-chain soluble HLA-G product has been described [43] and was obtained from Organon NV (Oss, the Netherlands). Bacterially expressed and refolded HLA-G was produced as described for HLA-E [73]. Refolded HLA-C has been described [40] and was obtained from P. Sun (NIAID, NIH).

### Immunostaining and confocal microscopy

Cells were allowed to settle on poly-L-lysine–coated two-well culture slides (BioCoat, BD, Bedford, Massachusetts, United States) for 30 min prior to fixation in PBS/4% paraformaldehyde. Nonspecific sites were saturated and cells were permeabilized for 30 min with PBS/10% normal donkey serum/0.5% Triton X-100. Cells were stained with the relevant primary antibody for 1 h and revealed with goat anti-mouse or goat anti-rabbit Alexa-Fluor–conjugated secondary antibodies in PBS/3% normal donkey serum/0.5% Triton X-100. In some cases, cells were stained with directly conjugated antibodies (e.g., 33-Cy3). Cells were washed and mounted in slides using the Prolong Anti-fade kit (Molecular Probes, Eugene, Oregon, United States).

Images were processed using a confocal laser-scanning microscope (Axiovert 200M LSM 510 META; Zeiss, Jena, Germany) fitted with a 1.3 Oil DIC Plan-Neofluar ×63 objective was used. Images were acquired using channel mode, multitrack acquisition with the main beam splitters HFT 488/543 for the excitation of gfp and Alexa-Fluor 568. Filter sets of BP 505–530 for gfp and LP560 for Alexa-Fluor 568 were used. Parameters were adjusted to yield scan control of fixed pixel density at 512 × 512 pixels, 12-bit pixel depth, and a pinhole size of 90 μm. To quantify the co-localization of KIR2DL4 with loaded soluble HLA-G, the LSM 510 imaging examiner software was used.

### Electron microscopy

The NK cell lines NKL and YTS-2DL4-gfp were loaded with KIR2DL4-specific Ab 33 for 2 h. Cells were rinsed in HBSS before fixation with periodate-lysine-paraformaldehyde (PLP) fixative containing 0.25% glutaraldehyde for 2 h at room temperature. Cells were permeabilized with PBS containing 0.01% saponin for 5 min at room temperature and incubated for 1 h with peroxidase-conjugated F(ab′)_2_ sheep anti-mouse IgG (Jackson Immunoresearch Laboratories, West Grove, Pennsylvania, United States) in PBS containing 0.01% saponin. Cells were rinsed in PBS and fixed in 1.5% glutaraldehyde in 0.1 M sodium cacodylate (pH 7.0) plus 5% sucrose for 1 h. Cells were then rinsed with 50 mM Tris-HCl (pH 7.4) plus 7.5% sucrose before development with Immunopure Metal–enhanced 3,3′-diaminobenzidine substrate (Pierce, Rockford, Illinois, United States). Cells were then rinsed three times with 50 mM Tris-HCl, (pH 7.4) plus 7.5% sucrose before fixation in 4% paraformaldehyde/2.5%glutaraldehyde in 100 mM sodium cacodylate buffer (pH 7.4). Cells were postfixed in 1% OsO_4_/1%K_3_Fe(CN)_6_, washed with H_2_O, dehydrated in a graded ethanol series, and embedded in Spurr's resin. Thin sections were cut with an MT-7000 ultramicrotome (RMC, Tucson, Arizona, United States). Samples were examined on a Hitachi H7500 TEM at 80 kV and images were captured with a CCD camera (Advanced Microscopy Technologies, Danvers, Massachusetts, United States).

### Cellular assays

For cytokine/chemokine assays, resting NK cells were incubated with soluble mAbs at 10 μg/ml, plate-coated antibodies (5 μg Ab/100 μl/well of a 96-well plate) or bead-bound antibodies (1 μg antibody/4 × 107 beads; four beads per cell). For soluble HLA-G stimulation of resting NK cells, recombinant single-chain HLA-G (30 μg/ml) was used together with either control IgG2a mAb or anti-HLA-G mAb G233 at 90 μg/ml. After the indicated timepoints, supernatants were removed and tested for the presence of cytokines by ELISA. ELISA kits for IL-1β, TNF-α, IL-6, IL-23, MIP-3α, IL-12, IL-5, and IL-13 (R & D Systems, Minneapolis, Minnesota, United States), IFN-γ (Pierce), and IL-8 (Biosource, Camarillo, California, United States) were used according to manufacturers' instructions.

For endocytosis assays, resting NK cells, 293T-2DL4-gfp, or YTS-2DL4-gfp cells were loaded with mAb 33 (10 μg/ml) or with 50 μg/ml of soluble HLA-G for the indicated time periods at 37 °C. Cells were then washed in PBS and plated onto chamber slides for immunofluorescence staining. For the blocking studies, mAb 33, KIR2DL4-Ig, or KIR2DL1-Ig (50 μg/ml) was added at the same time as soluble HLA-G.

Binding assays using Ig-fusion proteins were done essentially as described previously [8,74]. For blocking studies, mAbs to MHC molecules were added to the cells 20 min prior to the addition of KIR2DL4-Ig fusion proteins. KIR2DL4-specific mAb 33 was preincubated with KIR2DL4-Ig fusion proteins for 20 min prior to addition to the 221 cells.

For microarray analysis, NK cells were stimulated with either isotype control IgM antibodies (Sigma) or anti-2DL4 mAbs 36 and 64 for 5 h at 37 °C. Total RNA was purified from cell pellets by TriZOL/chloroform extraction followed by column purification (RNeasy Midi Kit; Qiagen, Valencia, California, United States). The integrity of isolated RNA was examined by denaturing TAE agarose gel electrophoresis. Direct labeling of first-strand cDNA with Cy3-dUTP or Cy5-dUTP (Amersham Pharmacia, Piscataway, New Jersey, United States), respectively, was performed in oligo(dT) primed reverse transcription reactions (SuperScript II Reverse Transcriptase; Invitrogen, Carlsbad, California, United States). The remaining RNA was degraded, and Cy3- and Cy5-labeled probes were combined and purified by buffer exchange on Vivaspin 500 columns (10K cutoff; Vivascience, Hannover, Germany). Labeled cDNA was hybridized to 14,000 oligonucleotide arrays (NIAID Microarray Facility, NIH). Arrays were scanned on an Axon GenePix 4000B scanner (Axon Instruments, Foster City, California, United States). Image analysis was carried out using GenePix (Axon Instruments) and mAdb (National Cancer Institute; http://madb.nci.nih.gov) software.

To investigate transcription of microarray candidate genes by RT-PCR, specific primers were designed to amplify regions of approximately 200 bp that spanned exon-intron boundaries. Total RNA was isolated by TriZOL/chloroform extraction followed by column purification (RNeasy Mini Kit; Qiagen). First-strand cDNA was synthesized with oligo(dT) primed SuperScript II reverse transcriptase (Invitrogen) from 600 ng of total RNA, according to the manufacturers' protocol. PCRs were performed with Taq polymerase according to the manufacturers' protocol in 25 μl reactions. First-strand cDNA template corresponding to 10 ng total RNA per reaction were used in touch-down PCRs with five cycles (annealing temperature 70 °C) followed by 20 to 35 cycles (annealing temperature 65 °C). Primers to *CCL20* (forward 5′- CCAATGAAGGCTGTGACATCAATG-3′ and reverse 5′- ACCTCCAACCCCAGCAAGGTTC-3′), *CXCL3* (forward 5′- TGCAGACACTGCAGGGAATTCAC-3′ and reverse 5′- CTTCTCTCCTGTCAGTTGGTGCT-3′), *GAPD* (forward 5′- GGCATGGACTGTGGTCATGAG>-3′ and reverse 5′- TGCACCACCAACTGCTTAGC-3′), *IFNG* (forward 5′- AGCGGATAATGGAACTCTTTTCTTAG-3′ and reverse 5′- AAGTTTGAAGTAAAAGGAGACAATTTGG-3′), *IL1B* (forward 5′- TCCAGGGACAGGATATGGAGCAA-3′ and reverse 5′- GCTTTTCCATCTTCTTCTTTGGGT-3′), *IL6* (forward 5′- TCGGTACATCCTCGACGGCATC-3′ and reverse 5′- ATACCTCAAACTCCAAAAGACCAG-3′), *IL8* (forward 5′- CTGATTTCTGCAGCTCTGTGTGA-3′ and reverse 5′- GGGTCCAGACAGAGCTCTCTTC-3′), *IL12B* (forward 5′- ACATTCTGCGTTCAGGTCCAGG-3′ and reverse 5′- CTCCAAATTTTCATCCTGGATCAG-3′), *IL23A* (forward 5′- ACTCAGTGCCAGCAGCTTTCAC>-3′ and reverse 5′- CAGACCCTGGTGGATCCTTTGC-3′), *MARCKS* (forward 5′- GAGCAAGCTTTTGTGAGATAATCG-3′ and reverse 5′- GAATGATTTGAGATGGGATCTGTG-3′), *PTGS2* (forward 5′- AATCATTCACCAGGCAAATTGCTG-3′ and reverse 5′- CTTCCAACTCTGCAGACATTTCC-3′), and *TNF* (forward 5′- CTCTTCTGCCTGCTGCACTTTGG-3′ and reverse 5′- CCATTGGCCAGGAGGGCATTGG-3′) were used.

PCR products were separated by horizontal agarose gel electrophoresis, stained with ethidium bromide, visualized with UV light, and photographed. Black/white images were inverted with Adobe Photoshop software (Adobe Systems, San Jose, California, United States).

## Supporting Information

### Accession Numbers

The GenBank (http://www.ncbi.nlm.nih.gov/Genbank/) accession numbers of the cDNAs used in this paper are KIR2DL4 (DQ266438) and HLA-G1 (M90683).

## Abbreviations

Ab: antibody
gfp: green fluorescence protein
HLA: human leukocyte antigen
HRP: horseradish peroxidase
IFN: interferon
IL: interleukin
KIR: killer cell immunoglobulin-like receptor
LAMP-1: lysosome-associated membrane protein 1
M6PR: mannose 6-phosphate receptor
mAb: monoclonal antibody
MHC: major histocompatibility complex
MIP: macrophage inflammatory protein
NK: natural killer
TNF: tumor necrosis factor
